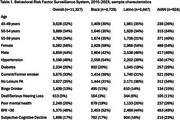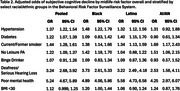# Midlife modifiable risk factors and subjective cognitive decline among Black, Latino and Native American/Alaska Native participants of the Behavioral Risk Factor Surveillance System

**DOI:** 10.1002/alz70860_107649

**Published:** 2025-12-23

**Authors:** Rachel Peterson, Lauren Cater

**Affiliations:** ^1^ University of Montana, Missoula, MT, USA

## Abstract

**Background:**

Black and Latino populations have well‐established disparities in dementia, while some studies also find higher rates among American Indian/Alaska Native (AI/AN). Less is known about the distribution of modifiable midlife risk factors across these groups and how these risk factors may predict self‐reported changes in thinking and memory (subjective cognitive decline).

**Methods:**

In the Behavioral Risk Factor Surveillance System (pooled 2015‐2023), we examined the distributions of dichotomized measures of hypertension, diabetes, ever smoking, leisure physical activity, binge drinking, deafness/severe hearing loss, depression, and having a BMI >30 among respondents ages 45‐59 who identified as Black, Latino or AI/AN. Logistic regression models estimated the odds of subjective cognitive decline corresponding with these risk factors overall and stratified by race/ethnicity. All models adjusted for sex and years of education.

**Results:**

We retained complete cases from 4,729 Black, 5,647 Latino and 924 AI/AN participants. Having a BMI>30 was the most prevalent risk factor overall (48%), followed by hypertension (46%) and no leisure physical activity (35%; Table 1). We observed substantial variability in prevalence of risk factors across racial/ethnic groups. The most prevalent risk factor among Black participants was hypertension (54%), among Latino participants was having a BMI<30 (44%) and among AI/AN was being a current/former smoker (56%). In pooled adjusted logistic regression models, all risk factors except binge drinking (OR=1.07 [95% CI = 0.91, 1.26]) predicted higher odds of subjective cognitive decline (Table 2). Poor mental health (pooled OR=5.24 [95% CI=2.67, 5.89]) and deafness/serious hearing loss (pooled OR=3.24 [95% CI=2.68, 3.92]) were the strongest predictors of subjective cognitive decline in both the pooled model and all race/ethnic stratified models. Among Black participants, the next strongest predictor of subjective cognitive decline was hypertension (OR=1.48 [95% CI=1.22, 1.79]). Among Latino participants, diabetes (OR=1.40 [95% CI=1.15, 1.70]) was the next strongest predictor. Among AI/AN participants, current/former smoker was the next strongest predictor (OR=1.69 [95% CI=1.17, 2.43]).

**Conclusion:**

The most relevant risk factors for subjective cognitive decline and promising targets for precision prevention of cognitive impairment are hypertension among Black Americans, diabetes among Latinos and smoking among AI/AN.